# Arkheia: Data Management and Communication for Open Computational Neuroscience

**DOI:** 10.3389/fninf.2018.00006

**Published:** 2018-03-05

**Authors:** Ján Antolík, Andrew P. Davison

**Affiliations:** ^1^Institut National de la Santé et de la Recherche Médicale UMRI S 968; Sorbonne Universits, UPMC Univ Paris 06, UMR S 968; Centre National de la Recherche Scientifique, UMR 7210, Institut de la Vision, Paris, France; ^2^Unité de Neurosciences, Information et Complexité, Centre National de la Recherche Scientifique UPR 3293, Gif-sur-Yvette, France

**Keywords:** computational modeling, workflow, publish, neuroscience, tool

## Abstract

Two trends have been unfolding in computational neuroscience during the last decade. First, a shift of focus to increasingly complex and heterogeneous neural network models, with a concomitant increase in the level of collaboration within the field (whether direct or in the form of building on top of existing tools and results). Second, a general trend in science toward more open communication, both internally, with other potential scientific collaborators, and externally, with the wider public. This multi-faceted development toward more integrative approaches and more intense communication within and outside of the field poses major new challenges for modelers, as currently there is a severe lack of tools to help with automatic communication and sharing of all aspects of a simulation workflow to the rest of the community. To address this important gap in the current computational modeling software infrastructure, here we introduce Arkheia. Arkheia is a web-based open science platform for computational models in systems neuroscience. It provides an automatic, interactive, graphical presentation of simulation results, experimental protocols, and interactive exploration of parameter searches, in a web browser-based application. Arkheia is focused on automatic presentation of these resources with minimal manual input from users. Arkheia is written in a modular fashion with a focus on future development of the platform. The platform is designed in an open manner, with a clearly defined and separated API for database access, so that any project can write its own backend translating its data into the Arkheia database format. Arkheia is not a centralized platform, but allows any user (or group of users) to set up their own repository, either for public access by the general population, or locally for internal use. Overall, Arkheia provides users with an automatic means to communicate information about not only their models but also individual simulation results and the entire experimental context in an approachable graphical manner, thus facilitating the user's ability to collaborate in the field and outreach to a wider audience.

## 1. Introduction

For most of its history, computational neuroscience has focused on relatively homogeneous models, targeting one or at most a handful of features of neural processing at a time. Such a classical reductionist approach is starting to be supplemented by more integrative strategies that utilize increasingly complex and heterogeneous neural network models, in order to explain within a single model instance an increasingly broad range of neural phenomena (Markram, 2006; Rangan et al., 2009; Markram et al., 2011; Koch and Reid, 2012; Bouchard et al., 2016; Hawrylycz et al., 2016). Even though the classical reductionist approach will remain important, an integrative research program seems unavoidable if we are to understand a complex dynamical system such as cortex (or the entire brain), whose computational power is underlined by the dynamical interplay of all its anatomical and functional constituents, rather than just their simple aggregation. Given its sheer scope and complexity, such an integrative research program is unlikely to succeed if implemented by individual scientists or even individual teams. Rather, a systematic incremental strategy relying on cooperation within the entire field will be required, whereupon new models build directly on previous work and all models are extensively validated against biological data and compared against previous models based on an increasingly exhaustive set of measures. These trends herald the shift of focus from model creation and simulation to model analysis and testing.

At the same time, this increasing need for collaboration within computational neuroscience is accompanied by a more general trend in science toward more open communication, both internally, with other potential scientific collaborators, and externally, with the wider public. Many examples have by now shown the value of such open science approaches (Anderson et al., 2002; Szigeti et al., 2014) to promote one's research and find new collaborations. Engagement of a non-academic enthusiast audience via open-science platforms can not only improve the public outreach of one's research program, but also contribute to the core scientific development. However, the effectiveness of such an opening up of one's research is critically dependent on the ease with which outsiders can engage with the exposed resources, which in turn critically depends on the quality of the (software) infrastructure used to serve said resources.

This multi-faceted development toward more integrative approaches and intensifying communication within and outside the field poses major new challenges for the software infrastructure available to computational neuroscientists. The set of tools involved in a typical modeler's workflow is expanding concurrently with growing complexity in the metadata flowing between them. Meanwhile the requirements for their efficient interfacing with the outside world (whether in the form of human users or other software tools) is growing. This growing complexity of the tasks involved in the typical modeler's workflow is putting strain on researchers, who are required to manage increasingly more complex software infrastructure while spending a substantial portion of their work-time either writing *ad-hoc* software solutions to cover poorly supported aspects of the workflow or handling them manually. This situation is clearly less than ideal, slowing down the pace of research while introducing errors and hindering its reproducibility.

The last four decades have seen numerous additions to the ecosystem of computational neuroscience tools, including efficient, well tested, and highly usable simulators such as Neuron, NEST, Brian, NENGO and others (Carnevale and Hines, 2006; Gewaltig and Diesmann, 2007; Bekolay et al., 2014; Stimberg et al., 2014), data management and parameter exploration tools such as Neo, Sumatra, Lancet, and Pypet and others (Davison et al., 2008; Stevens et al., 2013; Friedrich et al., 2014; Garcia et al., 2014; Sobolev et al., 2014; Meyer and Obermayer, 2016), neural data analysis toolkits SpikeViewer (Pröpper and Obermayer, 2013) HRLAnalysis (Thibeault et al., 2014), NeuroTools (http://neuralensemble.org/NeuroTools), Elephant (http://neuralensemble.org/elephant), and integrated workflow and simulation environments such as VirtualBrain, psychopy_ext, or Mozaik (Antolík and Davison, 2013; Kubilius, 2014; Woodman et al., 2014). Despite this rapid progress, the interfacing between the tools and communication with third parties (whether users or tools) remains limited, hindering the future development of integrative collaborative approaches in computational systems neuroscience. We identify the following aspects of the modeling workflow, all with implications for communication and interfacing, that are currently poorly supported and are key to resolving the outlined limitations of the present infrastructure:

1. Higher-level, flexible, modular model specification standards allowing for transparent and efficient communication and reuse of model components.
2. Exhaustive, explicitly formalized annotation of data generated during model simulation allowing for deep automatic introspection of the raw neural data in subsequent processing steps (i.e., 3 and 4).
3. Explicit formalization of experimental protocols and neural data analysis allowing for (a) automatic testing and comparison of the models, (b) their efficient communication and reuse, and (c) deep introspection of the results
4. Tools that can utilize 1, 2, and 3 to automatically communicate and serve all aspect of the modeler's workflow to the rest of the community and public to facilitate collaboration and outreach.

Recently we have made advances in addressing some aspect of points 1, 2, and 3 by the release of the Mozaik toolkit (Antolík and Davison, 2013), which allows us here to start approaching the limitation 4, by introducing Arkheia^1^. Arkheia is a web-based platform for data management and communication of computational modeling outcomes in systems neuroscience. It provides an automatic, interactive, graphical presentation of simulation results, the experimental protocols used, and interactive exploration of parameter searches, via a web browser-based application. Arkheia is focused on automatic serving of these resources with minimal (virtually no) manual input from users. Arkheia is written in modular fashion with a focus on future development of the platform. It follows the standard database-server-client design and is based around modern widely adopted web-based technologies (MongoDB for the database, Node.js and Express.js for the server, and AngularJS for the client). Currently, Arkheia is shipped only with a Mozaik backend, as at present this is the only published framework providing sufficient introspection of simulated neural data that can be automatically harvested for the presentation in Arkheia. The platform is, however, designed in an open manner, with a clearly defined and separated API for database access, so that any project can write its own backend translating its data into the Arkheia database format. This both allows any current private *ad-hoc* project to use Arkheia as its graphical book-keeping and publishing frontend, as well as ensuring Arkheia can be used with any future workflow tools that may be developed. Arkheia does not currently offer an internally implemented fine-grain access control and is not meant to be used as a centralized platform. It rather allows any user (or group of users) to set up a separate repository, either publicly for access by the general population, as well as locally for internal use.

Overall, Arkheia provides users with an automatic means to communicate not only their models but also individual simulation results and the entire experimental context in an approachable graphical manner to a wider audience. Arkheia thus addresses some of the limitations of the present computational neuroscience infrastructure in managing and communicating results, thus facilitating the user's ability to collaborate in the field and outreach to a wider audience.

## 2. Comparison to other tools

Several recent software projects have overlapping goals with Arkheia. Lancet (Stevens et al., 2013) and Pypet (Meyer and Obermayer, 2016) are Python simulation workflow libraries that provide users with a means to organize and automate their numerical simulation workflow, automate exploration of model parameter space and manage the resulting data. Similarly to Arkheia, they provide users with structured access to the data produced in the simulations, enriched with a limited set of metadata tracked during the simulation workflow, mostly comprising the parametric configuration of the simulated models. Arkheia does not provide direct handling of the simulation workflow or the exploration parameter space, these are instead expected to be performed by the source of the data handled by Arkheia, and the Mozaik toolkit for which the data-import backend is currently provided offers both these services. Crucially, the Lancet and Pypet toolkits do not provide a graphical interactive representation of the data to the user, which is the primary goal of Arkheia. Furthermore, Lancet and Pypet are agnostic as to the nature of the simulations they handle, which makes them more general, but at the cost of explicitly exposing only a very limited set of information about the simulations to the user. In contrast, Arkheia is focused exclusively on neural simulations allowing it to provide the user with much richer and deeper introspection of information about the simulations held in the repository and to do it via a clear and convenient web-based graphical user interface.

Probably the most similar tool currently available to the computational neuroscience community is the Open Source Brain (OSB) (www.opensourcebrain.org) project, a web-based open science collaborative platform aspiring to become the go-to repository for neural modeling projects that wish to open themselves up for collaboration with the rest of the academic community. OSB technology is built around the NeuroML v2 data format for neural model specification. OSB is not structured around single simulation runs but around projects (which loosely correspond to single models), for which it provides a web front-page listing some essential information (e.g., project description, members, references, etc. ) and link to the project's code repository (e.g., Github). For a project that is not converted to the NeuroML format this is all information that is directly introspectable from OSB. For such projects, OSB essentially provides a centralized space where model authors can build a web-page about their project. Additionally, for parts of the project that are converted to NeuroML one can invoke the Geppetto (www.geppetto.org) Java interface within the web-browser allowing the user to inspect the model in detail via a GUI. One limitation of this approach is that NeuroML has been designed for detailed morphological neural models, and large-scale point neuron simulations, common in systems neuroscience, are not as well supported. Unlike Arkheia, OSB does not offer an explicit formalized presentation of the stimulation, results, experimental protocols, and their parametric context, which we argue are key for further development of collaborative tools in computational neuroscience.

## 3. Architecture

Arkheia follows the standard database-server-client architecture. To facilitate efficient, but at the same time flexible, storage of complex highly structured data that describe the makeup and results of neural simulations we have selected a modern document based database MongoDB (www.mongodb.com). The nature of the data describing a simulation run and its results are straightforwardly described by a hierarchical document that can be efficiently represented and retrieved in a document-based database. Presently MongoDB is an industrial standard for document based databases and is particularly frequently used in web based software solutions. It is well-supported and accessible to new users, overall making it a suitable choice for this project.

**Figure 1:**
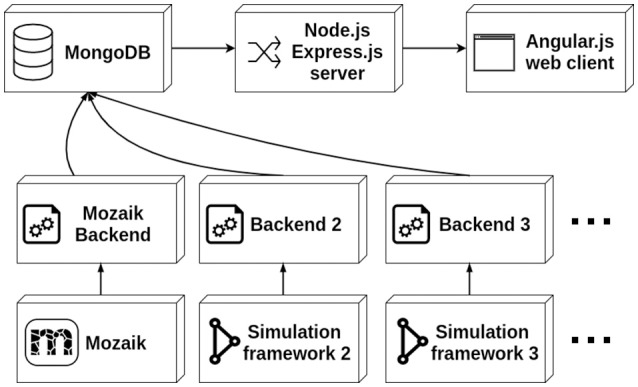
The architecture of Arkheia. The MongoDB database stores the information about individual simulation runs as documents. These are served via the thin server layer built on top of the Node.js platform using the Express.js web-server to the client, which is a multi-page AngularJS-based web-application. The backends directly connect to the database and insert documents that contain the description of a given simulation run. The backends are expected to automatically export this data from a given simulation framework.

The data stored in the database is served to the client via a thin server layer developed in the the asynchronous event driven JavaScript runtime Node.js (nodejs.org) using the Express.js (expressjs.com) web-server package. The client is a web application written in the AngularJS (angularjs.org) framework (see Figure 1). The server uses the Mongoose library to access and standardize the data stored in the MongoDB instance. The architecture of the client follows the Model-View-Controller (MVC) design facilitated by the AngularJS framework. Here the different Angular models map onto different parts of the simulation run description (i.e., the root list of simulation runs, stimuli, results, experiment protocols etc.), and each Angular model is associated with an HTML template and a controller handling the dynamic aspects of the views. The client is a multi-page web application offering multiple views of the simulation run data, mostly following a tabular presentation pattern. In addition a more complex web application for interaction exploration of parameter search results is offered.

The insertion of data into Arkheia is expected to be done by an arbitrary set of backends, which should automatically export data from a given simulation framework and insert it into the MongoDB database in the format expected by Arkheia, which is described in the following section. The backends connect directly to the database, and thus no assumptions about their behavior beyond the insertion of the data in the correct format are made. The interaction of Arkheia with external tools is thus fully specified by the format of data stored in the database. While the backends are primarily meant as automatic exporters from simulation environments, in principle one could create an interactive GUI based application (which from the point of view of Arkheia would behave as any other backend) that would allow manual insertion of data into Arkheia.

## 4. API

**Figure 2:**
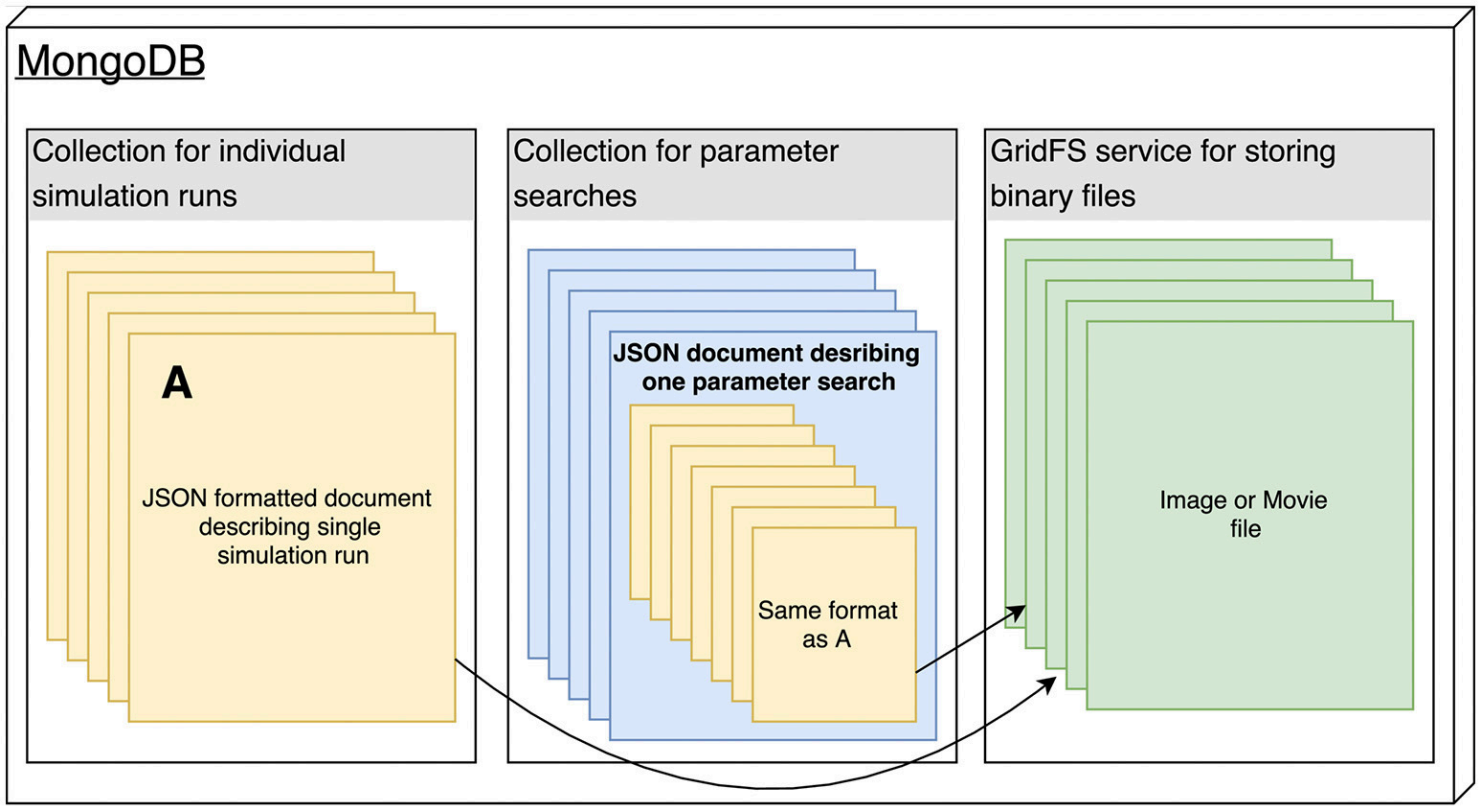
The architecture of the Arkheia MongoDB database. All data are stored in three MongoDB collections, one for the individual simulation runs, one for the parameter searches, and one optimized for storage of binary data using the GridFS service, which will store the images and movies that are referenced from the documents stored in the other two collections. Any html-permissible image or movie format is supported by Arkheia.

The Arkheia API is essentially a description of how the shared data about individual simulation runs should be stored in the MongoDB database used by Arkheia (see Figure 2). The data is stored in three MongoDB collections, one storing the individual simulation runs, one storing the parameter searches and one storing any binary files (e.g., images and movies) that are referenced from the documents in the other two collections (see Figure 2). Thus, with the exception of the mechanisms for storing of image and movie data, this storage description reduces to the description of the format of the hierarchical document that will be stored for each simulation run. This specification covers the storage of model specification, sensory stimuli, experimental protocols, resulting data analysis, and visualization outputs. We expect rapid development in the specifications of data Arkheia handles, both as the scope of Arkheia expands, but more importantly as we hope standardized specifications of some aspects of the data will develop in the field in the near future (Eglen et al., 2017).

### 4.1. Simulation run representation

Arkheia data specification follows a document based design, which conveniently maps onto the document based MongoDB database used by Arkheia. Each simulation run is represented by a single JSON hierarchical data-structure which corresponds to the document inserted into the database. Thus the Arkheia input data specification reduces to the expected format of this JSON data-structure. At the root level the SimulationRun data-structure contains the following entries with the indicated value types:


{
                 ’submission_date’               :   string,
                 ’run_date’                          :   string,
                 ’simulation_run_name’        :   string,
                 ’model_name’                    :   string,
                 ’results’                              :   list of Result,
                 ’stimuli’                              :   list of Stimulus,
                 ’recorders’                         :   list of Recorder,
                 ’experimental_protocols’    :   list of Protocol,
                 ’parameters’                       :   ParameterSet
}


The *submission_data* and *run_date* entries are expected to be strings representing time in “*YYYY/MM/DDHH:MM:SS*” format. The *simulation_run_name* is an arbitrary name given to this specific simulation run (not the model). The *model_name* is the name of the model that was simulated. The *results, stimuli, recorders*, and *experimental_protocols* are each a list of JSON data structures, the format of which will be described below. Finally the *parameters* variable should describe the full parametrization of the model used in this run, and as all parametrization throughout Arkheia API, it should follow the *ParameterSet* format.

*ParameterSet* is a nested dictionary (see schema below) where each value associated with a key (that corresponds to the name of the parameter) is a tuple (*a, b, c*), where *a* corresponds to the value of the parameter and can either be a scalar or *ParameterSet* itself, *b* is the type of the parameter, and *c* is a short description of the parameter's meaning.


ParameterSet = {
                 ’key’   :   (scalar value, scalar type,
                                description string),
                 or
                 ’key’   :   (ParameterSet, dict,
                    .           description string)
                    .
                    .
                    .
           }


The *results* entry should contain a list of Result JSON data-structures, each describing one result produced during the simulation (presumably after analysis and visualization of the raw data recorded during the simulation). Each result is meant to be represented as a figure with an accompanying explanatory caption, and is represented as the following JSON data-structure:


{
                 ’code’             :   string,
                 ’name’            :   string,
                 ’caption’         :   string,
                 ’parameters’   :   ParameterSet,
                 ’figure’            :   MongoDB GridFS ID
}


The *code* entry should contain a reference, for example a fully qualified class or function name, to the source code that generates the given figure. The *name* should contain the name of the figure and the *caption* should contain a caption that describes what is displayed in the figure. The *parameters* entry should contain the parameters with which the generator of the figure identified by *code* entry was invoked to generated the given figure, and should follow the *ParameterSet* format described above. Finally, the *figure* entry contains a reference to the image of the figure (in any format that is widely supported in browser) that was stored in the MongoDB GridFS file store.

The *stimuli* root level entry should contain a list of *Stimulus* JSON data-structures, each describing one of the stimuli presented during the simulation. It assumes that the model was presented with a list of stimuli, generated by some source code entity (class or function) and parameters, and its “raw” instantiation can be represented as a vector stream, which in turn can be translated into a movie for visual inspection by the user. The format of each *Stimulus* data-structure is as follows:


{
                 ’code’                       :   string,
                 ’short_description’    :   string,
                 ’long_description’     :   string,
                 ’parameters’             :   ParameterSet,
                 ’movie’                     :   MongoDB GridFS ID,
}


The *code* and *parameters* entries are analogous to those in the Result data structure. Entries *short_description* and *long_description*, respectively, should contain a brief (one sentence) and a more detailed description of the stimulus. The *movie* entry should contain a reference to the movie (animated gif) of the stimulus (stored in the MongoDB GridFS).

The *recorders* root level entry should contain information on the recording configuration present during the stimulation. It assumes that this can be described as a list of parametrized entities that each records some set of variables from some set of neurons. The *recorders* entry should thus contain a list of *Recorder* JSON data structures, each corresponding to one such recording configuration entity. The format of the *Recorder* data structure is as follows:


{
                 ’code’                      :   string,
                 ’short_description’    :   string,
                 ’long_description’     :   string,
                 ’parameters’             :   ParameterSet,
                 ’variables’                 :   list of strings,
                 ’source’                    :   string
}


The *code, parameters, short_description*, and *long_description* entries are analogous to those in the *Stimulus* data structure. The *variables* entry should contain a list of strings, each identifying the variable that the given recording configuration recorded in the selected neurons, and the *source* should contain the name of the population of neurons to which this recording configuration was applied.

Finally the *experimental_protocols* root entry is expected to contain a list of *Protocol* JSON data structures characterizing the experimental protocols that were performed during the simulation of the model. The format of each of the *Protocol* data structures is as follows and the semantics of all the parameters are analogous to the corresponding entries in the *Stimulus* data structure:


{
                 ’class’                      :   string,
                 ’short_description’   :   string,
                 ’long_description’    :   string,
                 ’parameters’            :   ParameterSet
}


### 4.2. Parameter search representation

Parameter search is essentially a collection of simulation runs with a systematically varying subset of parameters. In principle, it would be possible to add the simulation runs originating from parameter searches into the same database as the individual simulation runs, and keep a separate record of their membership to the parameter search group. We have decided against such an organization, as parameter searches can consist of hundreds or more runs and would thus clog the presentation of the individual runs, as well as potentially make access to the individual run database slow. The parameter searches are thus stored in a separate database (within the same instance of the MongoDB server). Each parameter search is a JSON document of the following format:


{
                 ’submission_date’                :   string,
                 ’name’                                 :   string,
                 ’simulation_runs’                  :   list of SimulationRun,
                 ’parameter_combinations’    :   list of tuples
}


Where *submission_date* is expected to be a string containing the data of the submission to the repository, the *name* entry is the name given to the simulation run. The *simulation_runs* entry should contain a list of *SimulationRun* JSON data-structures, each corresponding to one simulation run with the same format as the JSON data-structure describing an individual simulation run described in the previous section. Finally the *parameter_combinations* entry should contain a list of tuples, with each tuple holding the name of the parameter that was varied, and a list of parameter values that were explored (note that currently Arkheia supports only grid parameter searches, although irregular grids, and missing elements are both supported).

### 4.3. API design discussion

First we want to emphasize that the goal of Arkheia is not to provide storage for the full raw data associated with a given simulation, but rather a structured description of the simulation context and results, sufficient for replication or reuse of the various aspect of the given simulation, and to facilitate a convenient human readable comparison and browsing of models and their results.

The data format specification described above broadly follows the organization of data in the Mozaik framework, facilitating seamless integration with the workflow toolkit, but is designed with sufficient flexibility to allow integration with other modeling frameworks. The specification makes a number of assumptions about the nature of data describing the simulation run and its results, which, however, we believe are mappable onto the majority of use cases in systems neuroscience.

For example we assume that the full parameterization of the model can be expressed as a tree structure, or more precisely a forest, as we allow multiple root entries. Note that the most common format for simulation configuration, a plain list of parameter names and their values, is trivially mapped onto the forest structure as a forest of depth 1, while we are not aware of any major simulation configuration schemes that cannot be mapped straightforwardly onto such a tree structure. If the configuration involves binary data (e.g., a specific image used for stimulation, or some data derived from biological experiments that set specific neuron-to-neuron connectivity), these can be simply referenced by the name of the file containing the binary data, which can be later—if necessary —looked-up in an external repository holding the full raw data of the given simulation run.

Another general approach within the specification securing flexibility and generality is the assumption that the various entities to be shared are generated by well-defined blocks of code that can be easily referenced (i.e., classes or functions) and thus by sharing the reference to this code and the parametrization used in the given specific simulation run, one can fully recreate the entity providing one has access to the full source code of the given simulation framework (and any potential additions to it by the author of the simulation). Note that the latter is a reasonable assumption under the condition that the author is willing to publish the model in Arkheia and should in any case become a standard practice in the age of open science. These code entities should furthermore be accompanied by a detailed description of the given entity (e.g., experimental protocol) with an explanation of how the associated parameters configure the entity. An elegant solution to this is to keep this description as a code “docstring” of the given code block, where it fulfils the good practice of well documented code, and can be automatically harvested into the documentation of the given simulation framework while at the same time exported for publication in Arkheia.

The final source of flexibility is that most of the entries of the specification do not actually have to hold data (e.g., all the string values can be set to empty strings, or list values to empty lists). Arkheia can thus be used in projects which either do not use, or do not yet expose all the data so far covered in our specification. It is the intention that this specification will be developed further with the main goal of increasing the coverage of the information that can be exposed about simulation runs. We particularly hope that some of the ongoing efforts in the wider community will generate well-designed and popular specification standards for some of the aspects of neural simulations covered by Arkheia, such as standardization of experimental protocols, higher level model specifications or stimulus definitions, which we would eagerly seek to incorporate into Arkheia (Hucka et al., 2003). To this end Arkheia represents both a sketch of how such specifications could look and an example of how they could be used to facilitate communication and comparison across different models, thus motivating the development of these technologies, which we believe are key to the future computational neuroscience software infrastructure.

### 4.4. The back end implementation

The API presented above fully specifies the requirements Arkheia imposes upon its back-ends and thus all remaining implementation choices are left to the author of the given back-end. However, to provide reader a basic understanding of what it takes to implement such a back-end, we will in this section present a brief account of the Mozaik back-end that currently ships with Arkheia.

As a part of a simulation run, Mozaik creates a new directory, in Mozaik referred to as data-store, in which it stores the recorded neural signals coupled to a detailed specification of the simulation context. The role of the Mozaik Arkheia back-end is to simply transform this data-store (ignoring some aspects of it not covered in Arkheia such as raw neural signal recordings) into the Arkheia document format specified in this section above, and submit it into user specified Arkheia repository.

The Mozaik Arkheia back-end is written in *python* and uses the *pymongo* package to access and transmit the Arkheia document into the requested Arkheia repository, taking care of all the low level MongoDB related processing. Thus, the MongoDB specific code within the back-end amounts to only six lines of code (at the time of writing). We expect a similar level of complexity for other programming languages subject to availability of similarly functional MongoDB library.

The remaining work of the back-end is relegated to creating a nested python dictionary, restricted to elementary scalar types (i.e., float, int, string) or arrays of them as leafs, that follows the Arkheia document specification and reflects the information contained in the Mozaik data-store. Given the explicit exposure of all the required information in Mozaik, this amounts to simple systematic browsing through the data-store, using the access routines provided by Mozaik, and subject to few simple formatting translations re-inserting the information into the API pre-specified format. The one extra processing step the Mozaik back-end undertakes is that using the code-references (in the form of full path to generating class) of the various elements of the simulation context, it automatically harvests the values for the short and long description fields (see the API description above) from the “docstrings” of the referenced code. This is possible because of the docstring formatting convention [required by the Sphynx (www.sphinx-doc.org) documentation package] that Mozaik adheres to. This Mozaik specific design choice saves time by reusing the information entered by the user during documentation of the code also for the purpose of serving via Arkheia. All-in-all, the entire back-end is thus only very modest 300 lines of code including all the auxiliary routines.

In terms of usage, the Mozaik back-end is invoked via command-line, with two parameters: the path to the data-store previously created by Mozaik simulation run, and the address of the Akrheia repository to which the users wishes to submit the results. It should be noted that we have chosen this command-line centric usage for its flexibility, but in principle the invocation of the back-end could for example be directly integrated into Mozaik platform so that it automatically happens at the and of each simulation run, or integrated into any GUI, if available for the simulation platform. Ultimately this design choice is left to be made by authors of any specific back-end.

The amount of work required to implement a back-end will naturally vary between simulation platforms, depending mainly on how explicitly represented and easy to retrieve the various information that Arkheia serves are in the given simulation platform. However, here we demonstrate, that for those platforms built with the explicit coupling of *in-silico* recordings with the information about simulation context in mind, this can be a very straight-forward process.

## 5. Web based graphical frontend

Alongside the server specification, we have built a web-based application that serves as the visual front-end of the platform. The web application allows users to visually inspect the content of the data-store including the model specifications, visual stimuli, experimental protocols, and the outputs of model analysis and visualization. It also provides a means for interactive inspection of parameter search data, which tends to be an integral part of systems neuroscience modeling, but has so far lacked a dedicated interactive GUI based tool.

The Arkheia web-app is composed of multiple views with repeating design patterns, and so for the sake of conciseness and scientific relevance, we will describe only some of the key views, which will, however, offer a representative image of the application. For a complete run-through of the GUI the user can refer to the documentation provided with the Arkheia demo repository at http://arkheia.org. Upon landing on the Arkheia home page, the user is presented with basic, user-specified information about the given repository (note that it is expected that multiple Arkheia repositories will be deployed by different users on the web). Apart from navigating back to the landing page, and exploring the documentation, the top navigation bar offers links to the two main parts of the web application: simulation runs and parameter searches.

### 5.1. Individual run inspection

Most views of the Arkheia web app follow a tabular design, and the main simulation run view is a typical example. Each line of the table corresponds to one simulation that has been added to the Arkheia database. The first four columns offer basic information about the given simulation run, specifically the time of its submission to the repository (Figure 3.1), the approximate time when the simulation was executed (Figure 3.2), the label the user gave to the specific simulation run (Figure 3.3), and finally the name of the model that was simulated (Figure 3.4). The last five columns then contain links to other views that show more detailed information about the given simulations, specifically a pop-up that contains brief description of the model (Figure 3.5), a view showing the full parametrization of the given simulation (Figure 3.6), a view showing the stimuli presented to the model during the simulation (Figure 3.7), a view showing the experimental context of the simulation (Figure 3.8), and a view showing the results generated during the simulation (Figure 3.9). The last column allows users to download the entire Arkheia document together with the associated figure images as a zip file (Figure 3.10). Finally, the “search” icon in the top header allows users to filter the displayed simulation runs based on any attribute of the associated hierarchical Arkheia document (Figure 3.11). Refer to Arkheia documentation for more details on all the discussed functionality.

**Figure 3:**
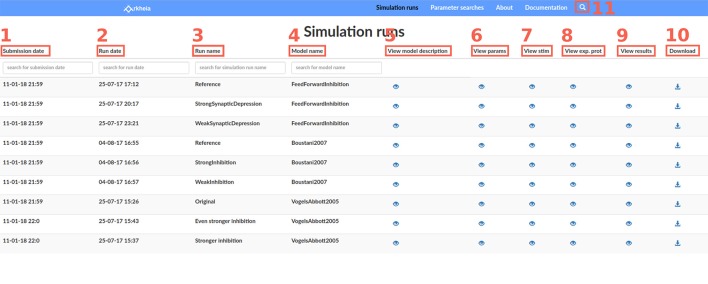
The Simulation runs view. Each row of the table corresponds to one simulation run which was added to the Arkheia repository. Each column corresponds to a different property of the simulation run. From left to right, the submission date **(1)** the approximate time when the simulation was executed **(2)**, the label the user gave to the specific simulation run **(3)**, the name of the model that was simulated **(4)**, a link that will bring up a pop with a brief description of the model **(5)**, a link to the view showing the full parameterization of the run **(6)**, a link to the view showing the stimuli presented **(7)**, a link to the view showing the experimental context **(8)**, a link to the view showing the results generated during the simulation run **(9)**, a link for downloading the corresponding Arkheia document **(10)**. User can also filter results based on any attribute within the hierarchical Arkheia document via the “search” icon in the header **(11)**.

The parameter view allows the user to explore the entire tree structure of parameters that fully specify the given simulation. A user can navigate through the tree using a classic tree-view interface, expanding and contracting nodes of the tree as desired (Figures 4.1,4.2). The leaves of the tree correspond to individual parameters, and contain their value or any other extra information that can be encoded into a human-readable string, such as the expected type of the parameter value (Figure 4.3).

**Figure 4:**
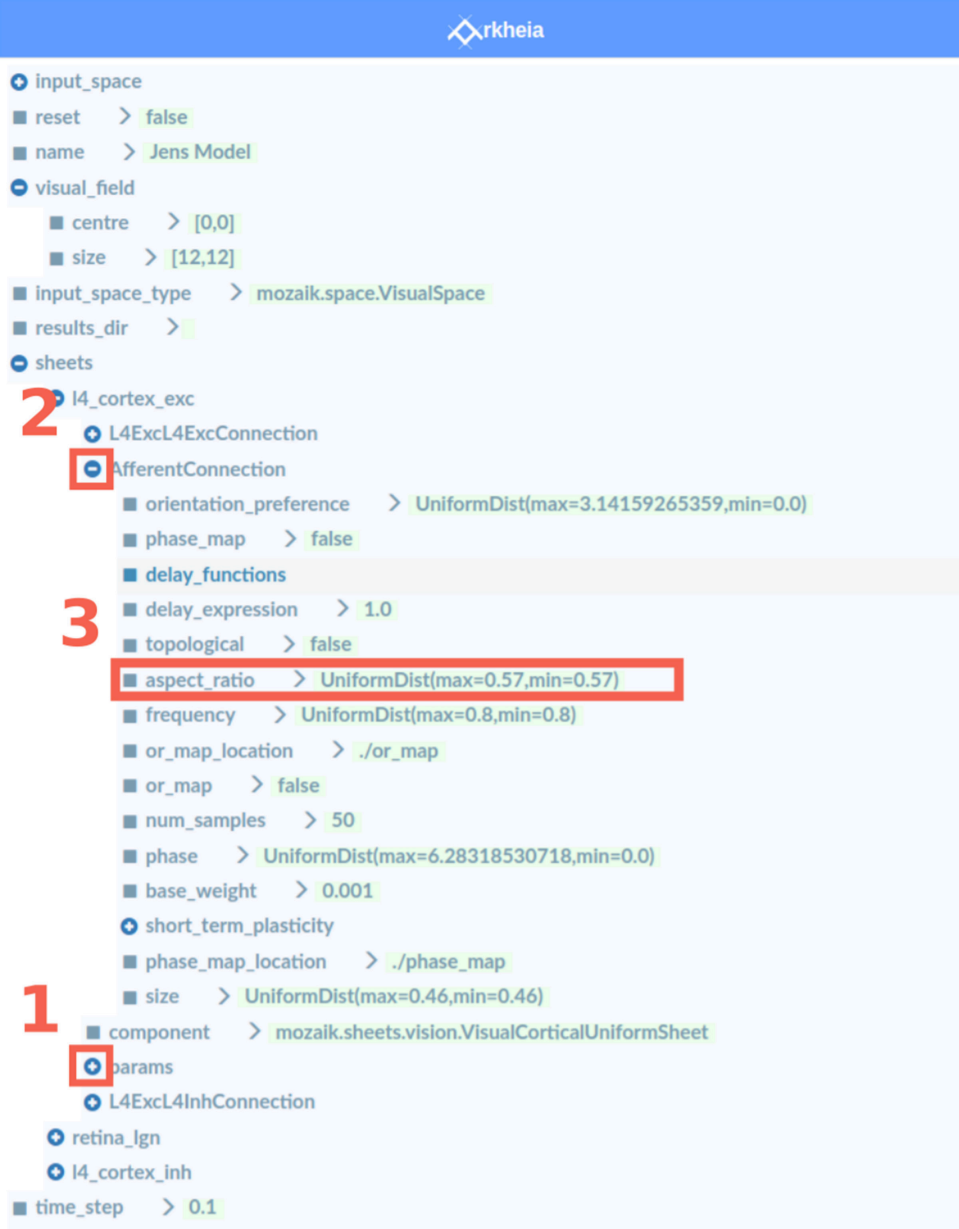
The Parameter view. This view contains a tree-view GUI element that displays and allows interactive exploration of the full parametrization of the simulation. The+ icons **(1)** indicate a folded node of the tree that can be further expanded upon selection, while the − icons **(2)** indicate an expanded node that can be contracted back upon selection of the icon. The leaves of the tree **(3)** then contain the values of the given parameter (that is specified by the full path from root to leaf). Arkheia is agnostic as to what the value is, it only expects a string, and so additional information, such as the parameter type, can be provided.

The stimuli, experimental context and results views all follow the same tabular design as the simulation runs. We will thus skip the description of the first two and only describe the latter. The results view contains the different results, in the form of figures, that were generated during the simulation, one per row. The first column contains identification of the code that was responsible for generating the given figure (Figure 5.1; typically a full path to a class or function is expected). The second column contains the name of the figure (Figure 5.2). The third column contains the thumbnails of the corresponding figures (Figure 5.3). These can be expanded to a full-sized view upon clicking the corresponding thumbnail. The full-size view panel also contains additional navigation elements with which the user can browse through the full size figures directly.

**Figure 5:**
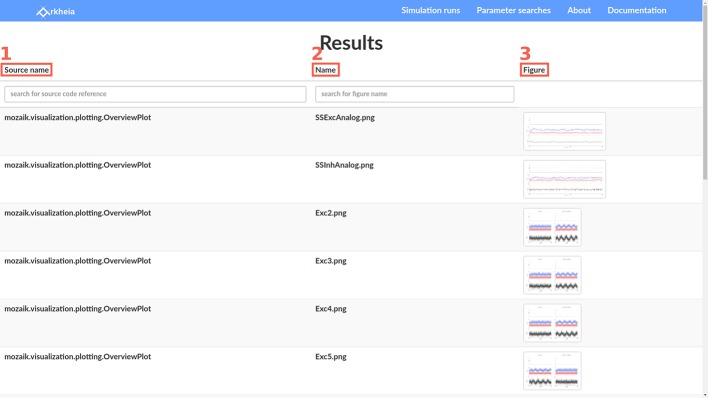
The Results view. Each row of the view corresponds to a figure that was generated during the simulation. The first row **(1)** shows a reference to the code responsible for the given figure. The second row **(2)** shows the name of the figure. The third **(3)** row shows thumbnails of the figures. Upon clicking a thumbnail the full sized figure is displayed.

### 5.2. Parameter search inspection

The augmenting complexity of models explored in neuroscience implies an increasing number of parameters, not all of which can be fully constrained a-priori by experimental data. This is particularly problematic in network models that typically have too many parameters and have too complex dynamics to be effectively fitted directly to data via optimization techniques such as gradient descent. This poses challenges for modelers, both in terms of finding values for the free parameters that induce the desired model behavior, and for generally understanding how the model behaves within its high-dimensional parameter space. The standard method for dealing with this situation is to identify some very small number of key parameters (ideally two), condense the model properties of interest into a scalar variable (e.g., mean rate across neurons in the network, or error function expressing proximity of the model properties to experimental data), perform a grid search within the selected parameter space and subsequently plot the resulting mapping from n-dimensional (usually *n* = 2) space to 1D space. However, in complex models with highly non-linear behavior it can be difficult to find such scalar measures of model performance, and a more general exploration of the model behavior as a function of its parameters is thus increasingly necessary. However, currently there is a lack of software tools to facilitate such an exploratory process. To this end we have extended Arkheia with a graphical tool for parameter dependent exploration of arbitrary model properties, as long as they can be expressed as a figure.

As the entry parameter searches page has a similar tabular organization to the individual simulation run view, we will skip its description here. For each parameter search entered into the repository, the user has two options. If user wants to inspect a single specific run in a given parameter search, user can select the provided link in the Arkheia column and corresponding row, which will transfer him/her to a page containing a list of all the individual runs in the parameter search. This page and all subsequent navigation is identical to the Simulation run page except that the list of simulation runs displayed here corresponds to the individual simulation runs in the selected parameter search. This design facilitates efficient development of the application as any changes to the individual simulation run presentation are automatically transferred also to inspection of individual simulation runs from parameter searches.

**Figure 6:**
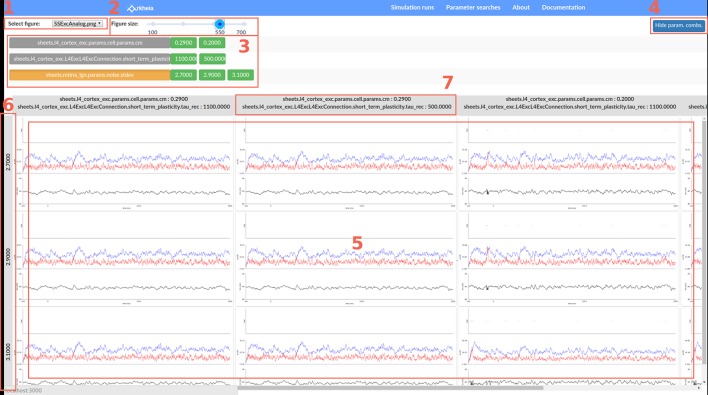
Interactive parameter search inspection in Arkheia. This page allows users to browse results of a parameter search of a given model. In the top left **(1)** the user can pick which of the figure types, generated in each of the simulation runs (i.e., a simulation run for a specific combination of parameters), to explore as a function of parameter values. In the top middle **(2)** the user can adjust the size at which the figures will be displayed. Below in the middle panel **(3)** is a list of parameters and their values that were explored. Here the user can activate/disable certain parameter values to explore only partial views of the data. The panel **3** can be hidden to gain more screen space for the figure grid by clicking button **(4)**. The bottom, largest panel of the page contains the grid of figures (**5**; filtered based on the selection made in the middle panel) with guides on the left **(6)** and top **(7)** showing the corresponding parameter combinations. User can scroll through this grid to interactively explore the data. Upon selecting any figure a full-screen version of it will be displayed for rapid detailed inspection.

The second, more interesting option, is to explore the entire parameter search via a grid based GUI. Upon selecting a parameter search to inspect, the user lands on a page depicted in Figure 6, which allows him/her to see a specific output of the parameter search (in the form of the selected type of figure) as a function of the parameter values that were varied in the parameter search via a grid-like interface (Figure 6.5). The grid provides two axes along which the parameter values can vary. The vertical axis always represents only one of the parameters (Figure 6.6), while the horizontal axis can correspond to combinations of parameters (Figure 6.7; or specifically their values), if more than two parameters were varied in the given parameter search. The parameter values are sorted along both grid axes so the user can discern how the properties of the model depicted in the selected figure change as a function of the different parameters and their values. The grid itself (Figure 6.5) then displays for each model parametrization a single figure (the identity of which is selected in Figure 6.1) at the associated grid position that was generated by the simulation. This makes the parameter search inspection interface general as it allows arbitrary model properties to be investigated as a function of the parameter changes, as long as they can be represented as an image (figure). Arkheia supports irregular spaced grids and missing entries, which are simply replaced with blank space in the grid view.

Above the figure grid, there is a panel listing all parameters that were varied in the parameter search (parameters of the models that were not varied will not be shown) and next to them all the values that the given parameter assumed in the search are displayed (Figure 6.3). By selecting a parameter, the user can choose which of the parameters should be displayed along the vertical axis of the grid (the remaining parameters will vary along the horizontal axis). Furthermore, the user can select any parameter value to exclude it from the display. This way the user can restrict the display of the parameter search to slices of the n-dimensional space of the varied parameters. This is a powerful method for gaining insight into how models behave with respect to their high-dimensional parameter space. Finally, at the top, the user can select which of the figure types generated during the simulation runs to view (Figure 6.1), as well as change their size (Figure 6.2).

## 6. Deployment

We envision two main types of deployment for Arkheia. First local, where a user utilizes Arkheia on a daily basis as a local store for his/her simulation results with the advantage of GUI access to them, and as a tool for interactive exploration of parameter search results. Second, public online deployment, where the Arkheia instance is used as a publishing platform for a user's models and results, for example accompanying publication with a much more detailed view of the data as well as a more thorough and usable specification of the model and entire experimental context. To provide an easy solution for both scenarios we offer several options for deployment of Arkheia.

In the simplest case, a user can install the two dependencies: Node.js and MongoDB. Clone the Git repository (https://github.com/antolikjan/Arkheia) of the project, run the MongoDB client, install the remaining dependencies by issuing *npm install* and *bower install* commands. Upon completing this user can issue the *gulp serve* command in the root directory, at which point user has running a local Arkheia instance at localhost:3000. New results can be added by pointing the given backend to the database. This setup is suitable for development, whereupon any changes to the Arkheia code will be automatically reflected in the web-browser. This is a very straightforward installation procedure, with narrow margin for error.

To facilitate even simpler deployment, especially in the cloud, we also provide a Docker (www.docker.com) image of the latest stable version of the system, with accompanying docker-compose specification which is configured for deploying the system on a server for public access. For example, on the DigitalOcean cloud service, the deployment via Docker reduces to selecting the “Docker” server type (i.e., “droplet”) in the DigitalOcean interface, copying the docker-compose (which is a tiny text file) specification, and issuing the *docker-compose up* command. With this extremely simple set of commands one now has an online Arkheia service accessible to the public. A similar simple deployment process, subject to any specifics of the other platforms, can be expected from any cloud service providers supporting Docker. Finally, the same process can be used on a private server, subject to the installation of the Docker service.

## 7. Future work

While the Arkheia repository as presented here already represents a fully functional neural simulation data management and publishing tool, we foresee a number of future improvements to the system. Among the different possibilities is the expansion of the platform to provide more fine-grained access to the analysis results and their visualization.

To further facilitate the outreach and collaboration goals of Arkheia, extension of the platform for multi-user use, and addition of multiple communication features, including forums, interactive chat, public outreach web page, would be beneficial. Alternatively, embedding Arkheia into a more general open science framework, such as Open Science Framework (osf.io) or HBP Collaboratory (Senk et al., 2017) would secure such communication features as well as many others.

## Author contributions

JA: Designed and implemented the presented software and wrote the manuscript; AD: Contributed to the design of the software and writing of the manuscript.

### Conflict of interest statement

The authors declare that the research was conducted in the absence of any commercial or financial relationships that could be construed as a potential conflict of interest.

## Footnotes

^1^Arkheia can be downloaded at https://github.com/antolikjan/Arkheia. A demo installation can be found at http://arkheia.org.
